# Association of quality of prenatal care with contraceptive planning in a United States population: a retrospective cohort study

**DOI:** 10.1186/s12905-023-02368-2

**Published:** 2023-05-02

**Authors:** Hannah L. Chapman, Dana Chase, Bikash Bhattarai, Maureen Sutton, Isuzu Meyer, Caleb Schofield

**Affiliations:** 1 Department of Obstetrics, Gynecology & Women’s Health, District Medical Group, Valleywise Health System, 2601 E Roosevelt St Phoenix, Phoenix, AZ 85008 USA; 2 Department of Obstetrics and Gynecology, University of Alabama at Birmingham, 1700 6th Avenue South, Birmingham, USA

**Keywords:** Prenatal care, Contraception plan, Family planning, Long active reversible contraception

## Abstract

**Background:**

Understanding how prenatal care influences planned postpartum contraception can help guide shared decision-making. This study looks to examine the association of the quality of prenatal care with planned postpartum contraception.

**Methods:**

This is a retrospective cohort study conducted in a single tertiary, academic urban institution in the southwest United States. The institutional review board (IRB) for human research at Valleywise Health Medical Center approved this study. Using a validated measure of prenatal care, the Kessner index, prenatal care was classified as adequate, intermediate, or inadequate. The World Health Organization (WHO) protocol for contraceptive effectiveness was used to classify contraceptives as very effective, effective, and less effective. The planned contraceptive choice was determined at the time of hospital discharge after delivery by discharge summary. Chi-squared testing and logistic regression were used to measure associations between the adequacy of prenatal care and contraceptive planning.

**Results:**

This study included 450 deliveries, 404 (90%) patients with adequate prenatal care, and 46 (10%) patients without adequate (intermediate or inadequate) prenatal care. There was not a statistically significant difference in planning for very effective or effective methods of contraception at hospital discharge between adequate (74%) and non-adequate (61%) prenatal care groups (p = 0.06). There was no association between the adequacy of prenatal care and the effectiveness of contraceptive planning after controlling for age and parity (aOR = 1.7, 95% CI 0.89–3.22).

**Conclusions:**

Many women chose very effective and effective methods of postpartum contraception; however, there was no statistically significant association between the quality of prenatal care and planned contraception at hospital discharge.

## Background

According to the American College of Obstetricians and Gynecologists (ACOG), approximately 45% of pregnancies are unintended and over one-third of pregnancies are short-interval, defined as conceived within 18 months of a previous birth [1]. Women plan some short-interval pregnancies, however, there are health benefits for women and their families through increased birth spacing [2]. Unintended and short-interval pregnancies are associated with risks including anemia, preterm birth, preeclampsia, and low birth weight infants [3, 4]. Furthermore, there are increased rates of postpartum depression and lower rates of breastfeeding for unintended pregnancies [3].

Peripartum contraceptive planning is imperative to prevent unintended and short-interval pregnancies. Antepartum and immediately postpartum are critical times for providers to initiate conversations about family planning and choice of contraception [5]. The Contraceptive CHOICE study was a prospective cohort study where there was standardized counseling on contraceptive choices, including long-acting reversible contraceptive (LARC) methods. Two-thirds of women chose LARC methods after standardized counseling and once financial barriers were removed [6]. The prenatal period is an important time for contraceptive counseling and shared decision-making. However, barriers remain for women to access their desired contraception and little research has evaluated the effect that the quantity of prenatal care has on these decisions and overcoming these barriers [7–9].

ACOG recommends initiation of prenatal care prior to 12 weeks’ gestation, with visits every 4 weeks until 28 weeks’ gestation, followed by visits every 2 weeks until 36 weeks’ gestation, then weekly visits until delivery [10, 11]. The current literature does not clearly identify a superior index for prenatal care utilization. However, the Kessner index is widely used in the literature because it contains validated measures and aligns with ACOG recommendations.

The primary objective of this study was to assess if there is an association between the adequacy of prenatal care and the efficacy of planned postpartum contraception at the time of hospital discharge after delivery. We hypothesize that those with adequate prenatal will have more opportunities for counseling and shared decision-making, and thus be more likely to plan for a very effective or effective form of contraception.

Methods:

This was a retrospective study conducted at a single tertiary, academic urban institution in the United States. The institutional review board (IRB) for human research at Valleywise Health Medical Center approved this study. All methods and data collection were carried out in accordance with IRB guidelines. The hospital serves a primarily Latina population and provides care through a system of community satellite clinics located throughout the county. Within this health system, there is a main campus hospital that offers the most comprehensive and intensive medical services including labor and delivery. The satellite clinics offer basic prenatal care services in less densely populated areas. Basic prenatal care is available at the satellite clinics which allows patients to get care during pregnancy closer to home and reduces the need to travel for prenatal care visits.

The study population was patients who received prenatal care at this institution and associated satellite clinics and delivered at the medical center between 1/2016 and 1/2017. Inclusion criteria were women aged 18 years and older who delivered at greater than 34 weeks’ gestation and had at least one prenatal visit at the institution or satellite clinic prior to delivery. Exclusion criteria included age less than 18 years at delivery, delivery at less than 34 weeks’ gestation, no prenatal care, and prisoners who delivered at the institution.

The current literature does not clearly identify a superior index for prenatal care utilization. The Kessner index and The Adequacy of Prenatal Care Utilization Index (APNCUI) have the strongest evidence regarding validity, predictability, and reliability [12]. Both also seem to align, but the Kessner index more so with regards to initiation of care, with current ACOG recommendations [13]. The Kessner index is utilized in this study given its wide use, validated measures, and alignment with ACOG.

Prenatal care was classified using the Kessner index based on the number of visits throughout the pregnancy and the trimester in which they initiated care. Prenatal care was classified as (i) adequate if it began in the first trimester and included nine or more visits for a pregnancy of 36 or more weeks, (ii) intermediate if it began in the second trimester or included five to eight visits for a pregnancy of 36 or more weeks and (iii) inadequate if it began in the third trimester or included four or fewer visits for a pregnancy of 34 or more weeks [12, 13]. For the current study, the subjects were compared between having adequate prenatal care versus non-adequate prenatal care (intermediate or inadequate).

The planned postpartum contraception choice was obtained from the hospital discharge summary. In this institution, there is universal screening for the postpartum contraceptive plan during prenatal care and contraceptive counseling during hospitalization as part of routine postpartum care. However, contraceptive counseling was not standardized during this study. Those women desiring contraception with postpartum tubal ligation, immediate post-placental IUD, implants, and Depo-Provera obtained their desired methods before discharge. Those who desired combined oral contraceptive pills, progestin-only pills, combined patches, and combined vaginal rings were provided with prescriptions and instructed on when to start their preferred method. If interested, those women who were uninsured at the time of delivery were eligible for tubal ligation, immediate post-placental IUD, implants, and Depo-Provera through grant funding at a reduced cost. The only barriers to obtaining any of these methods were specific insurance restrictions or the 30-day waiting period between obtaining consent and the Medicaid sterilization procedure.

Contraceptive choices were grouped by the WHO protocol for contraceptive effectiveness by dividing family planning methods based on the rates of unintended pregnancies per 100 women as the methods are commonly used by patients [14]. Contraceptive methods classified as very effective (0-0.9 unintended pregnancies per 100 women) include implants, vasectomy, female sterilization, levonorgestrel IUD, and copper IUD. Contraceptive methods classified as effective (1–9 unintended pregnancies per 100 women) include Depo-Provera injections, combined oral contraceptive pills, progestin-only pills, combined patches, and combined vaginal rings. Methods classified as less effective (> 10 unintended pregnancies per 100 women) include male condoms, diaphragms, fertility awareness, withdrawal, spermicides, no method, and other [14]. For the current study, very effective and effective contraceptives were compared to less effective contraceptives.

The medical records of the first 450 consecutive women who delivered at the institution from 1/2016 to 1/2017 and met inclusion criteria were reviewed. Patient demographic and clinical information abstracted from the electronic medical record included adequacy of prenatal care, planned postpartum contraception, age, parity, race, ethnicity, insurance, marital status, and mode of delivery. Given the limited data available regarding rates of contraceptive effectiveness as it relates to the level of prenatal care in the literature at the time of the study, a sample size estimation was not performed. Descriptive characteristics for demographic factors and adequacy of prenatal care analyses were conducted using Wilcoxon rank-sum tests for continuous variables, chi-square, or Fisher’s exact tests for categorical variables as appropriate. Binary logistic regression for the primary outcome of very effective or effective contraception controlling for age and parity. Statistical significance was set at 0.05. All statistical analyses were performed using SAS version 9.4 (SAS Institute Inc. Cary, North Carolina).

## Results

Overall participants (450 women) had a median age of 29 years (range 18–48), and most were Hispanic (79%). Of the 450 deliveries, 317 (70%) were uninsured, 115 (26%) had Medicaid, and 18 (4%) were privately insured. There were 227 (50%) single patients, 189 (42%) married patients, 27 (6%) domestic partnerships and 7 (2%) divorced patients (Table 1).

**Table 1:** Demographic characteristics of women included in study

Characteristics	Total (n = 450)	Adequate Prenatal care (n = 404)	Inadequate Prenatal care (n = 46)	p-value
Age (y), median, (range)	29 (18–48)	29 (18–48)	27.5 (18–40)	0.06
Parity, median, (range)	2 (1–9)	3 (1–9)	2 (1–6)	0.17
Race				
White	375 (83.3%)	340 (84.2%)	35 (76.1%)	0.39
Black	50 (11.1%)	42 (10.4%)	8 (17.3%)	
Asian	14 (3.1%)	13 (3.2%)	1 (2.2%)	
Native American	3 (0.7%)	2 (0.5%)	1 (2.2%)	
Other	8 (1.8%)	7 (1.7%)	1 (2.2%)	
Ethnicity				
Hispanic	356 (79.1%)	323 (79.9%)	33 (71.7%)	0.39
Non-Hispanic	93 (20.7%)	81 (20.1%)	13 (28.3%)	
Insurance				
Public (AHCCCS)	115 (25.6%)	102 (25.3%)	13 (28.3%)	0.75
Private	18 (4.0%)	17 (4.2%)	1 (2.2%)	
Uninsured/self pay	317 (70.4%)	285 (70.5%)	32 (69.5%)	
Marital status				
Single	227 (50.4%)	202 (50.0%)	25 (54.4%)	0.89
Married	189 (42.0%)	171 (42.3%)	18 (39.1%)	
Living w/ partner	27 (6.0%)	25 (6.2%)	2 (4.3%)	
Divorced	7 (1.6%)	6 (1.5%)	1 (2.2%)	
Mode of delivery				
Vaginal, spontaneous	334 (74.2%)	298 (73.8%)	36 (78.4%)	0.77
Vaginal, operative	19 (4.2%)	17 (4.2%)	2 (4.3%)	
Cesarean section	97 (21.6%)	89 (22.0%)	8 (17.3%)	

Of the 450 women, 404 (90%) had adequate prenatal care and 46 (10%) had non-adequate prenatal care (either intermediate or inadequate prenatal care). There were 21 (4%) with intermediate prenatal care and 25 (6%) with inadequate prenatal care. In the adequate prenatal care group, the mean number of prenatal care visits was 9. In the intermediate prenatal care group, the mean number of prenatal care visits was 4, and in the inadequate prenatal care group the mean number of prenatal care visits was 3. Due to the lower number of women in the intermediate and inadequate prenatal care groups and the mean number of prenatal care visits being 4 and 3 respectively, the subjects were compared between having adequate prenatal care versus non-adequate prenatal care (intermediate or inadequate). These groups did not have statistically significant differences in terms of age, parity, race, ethnicity, insurance, marital status, or mode of delivery (Table 1).

At the time of hospital discharge, 349 (78%) of patients had a contraception plan. The methods of contraception identified at hospital discharge from delivery included 151 (34%) opting for depo Provera, 100 (22%) for tubal ligation, and 28 (6%) for oral contraceptive pills, among others. Additionally, 101 (22%) patients planned for no method of postpartum contraception. Those without a planned contraceptive method were grouped in the less-effective contraceptive group (Table 2). There were 114 (25%) patients lost to follow-up at the postpartum visit.

**Table 2:** Contraceptive plan at hospital discharge grouped by effectiveness

Contraceptive Tier			
	Contraceptive Method	Frequency	Percent
Very effective		127	28.2%
	Implant	6	1.3%
	Vasectomy	7	1.6%
	Tubal ligation	100	22.2%
	Progesterone IUD	13	2.9%
	Copper IUD	1	0.2%
Effective		200	44.5%
	Depo Provera	151	33.6%
	OPCs	28	6.2%
	Progestin-only pills	20	4.5%
	Combined patch	1	0.2%
Less effective		123	27.3%
	Condoms	22	4.9%
	No method	101	22.4%
		450	100%

There was not a statistically significant difference in planning for very effective or effective methods of contraception between adequate (74%) and non-adequate (61%) prenatal care groups (p = 0.06, Table 3). There was no statistically significant association between the level of prenatal care and effectiveness of contraceptive planning after controlling for age and parity (adjusted OR = 1.69, 95% CI 0.89–3.22, Table 4).

**Table 3:** Association between prenatal care and contraceptive plan at hospital discharge

		Prenatal	Care			
		Adequate, n = 404,(%)	Non-adequate, n = 46, (%)	Total n = 450	Odds ratio (95% CI)	p
**Postpartum Contraceptive**	Very effective + effective	299 (74%)	28 (60.9%)	327	1.83 (0.98–3.45)	0.06
	Less-effective	105 (26%)	18 (39.1%)	123		

**Table 4:** Association between prenatal care and contraceptive choice controlling for age and parity

	Adjusted Odds ratio	95% CI
Contraceptive: Effective/very effective vs. Less effective	1.69	0.89–3.22
Age	1.04	0.99–1.10
Parity	1.04	0.83–1.30

## Discussion

There was not a statistically significant association between the effectiveness of contraceptive planning and the adequacy of prenatal care in the current study. This may have been due to the heavily weighted proportion of patients with adequate prenatal care compared to non-adequate prenatal care. However, there are other barriers to planning for and obtaining all forms of contraception, but especially highly effective postpartum contraceptives and this is an important area for future research and improvement in care [7–9].

Routine prenatal care involves expanded health screenings, preventative measures, directed treatments, and counseling with the aim to improve health outcomes for mothers and newborns [10, 11]. It also provides a unique window into the long-term health of women. There is currently no consensus on the best index to quantify the utilization of prenatal care. However, contraceptive and family planning are an integral part of prenatal counseling and treatment planning in terms of reproductive justice, patient autonomy, and prevention of unintended pregnancies [9, 10].

Multiple prior reports have demonstrated a disconnect between a woman’s interest in LARC methods during prenatal care and subsequent use in the postpartum period [7–9]. Although LARC methods are convenient and highly effective, they are often harder to access due to cost, planning for multiple clinic visits, additional testing, and misconceptions regarding their use and side effects [15]. Other barriers include insurance regulations that impact contraception by limiting the placement of LARC methods postpartum, requiring a 30-day waiting period between obtaining consent for Medicaid sterilization and performing the surgery and limited insurance coverage of postpartum contraception [8]. The same barriers that may lead to having limited access to prenatal care could also be barriers to sterilization and LARC methods.

Due to the 30-day waiting period between obtaining consent and the procedure for Medicaid sterilization, women with limited prenatal care who are interested in sterilization may be unable to access this method in the immediate postpartum period as they do not have the appropriate consent signed in advance [16]. Equitable access to all methods of contraception and sterilization regardless of insurance type is an important strategy to ensure patient-centered care.

Adverse maternal, fetal, or neonatal outcomes during prenatal care or intrapartum may influence the decision to adopt certain contraceptive methods. One study found that high-risk maternal conditions were associated with the intention to use and uptake very effective methods of contraception [17]. Another study found that following obstetric anal sphincter injuries, most women desired to wait 1 to 2 years before attempting another pregnancy. However, 41% of women in this group were not using any type of contraception at 3 months postpartum and one-third were using an effective or very effective method of contraception [18]. This discrepancy between desired birth spacing and contraceptive use highlights an important area for counseling to meet each patient’s family planning goals.

Additionally, efficacy is only one component of contraceptive decision-making. Complex social, cultural, and method-specific factors also influence this decision [19]. During shared decision-making, it is important to discuss all contraceptive options, and review their individualized risks and benefits, while considering each woman’s reproductive life plan, medical co-morbidities, and desires [20]. Patient-centered counseling and autonomy are critical to these discussions because women experience barriers to obtaining all forms of contraception as well as abortion in the United States [21]. Women who encounter these barriers to obtaining their preferred method of contraception are more likely to become pregnant less than 24 months after a delivery [9]. There can be a reduction in the number of unintended pregnancies if patients have access to quality contraceptive counseling and their desired method in the immediate postpartum period [22].

The strengths of this study included capturing a large patient population of women with varying levels of prenatal care. Limitations included the retrospective design, which limited information which could be abstracted from the medical record. The retrospective nature also limits the ability to infer causation, only association. There were no documented rates of contraceptive effectiveness by the level of prenatal care in the literature at the time of this study, so we did not perform a power calculation, which represented a convenience sample. There was universal screening for the postpartum contraceptive plan during prenatal care and contraceptive counseling provided during hospitalization as part of routine postpartum care. However, this counseling was not standardized or controlled for in the analysis. This is a limitation of the study and a potential area for care improvement.

Most participants included in this study had adequate prenatal care (90%), were Hispanic (79%), and were uninsured/self-pay (70%). These population characteristics limit the generalizability of our findings. However, given a lack of representation from minority ethnicity groups and uninsured individuals in clinical literature, the study adds to the current literature in these groups. Limitations of this study included that there was a large population with no prenatal care delivering at this hospital which was excluded from the study. We did not have the levels of education for this cohort so could not examine if this impacted contraceptive planning or the adequacy of prenatal care. Furthermore, we did not examine if there were associations between adequacy of prenatal care and initiation of contraception in the hospital. We also did not examine if there were associations between adequacy of prenatal care and contraception plan at the 4–6-week postpartum visit due to the large pool of patients, 114 (25%) who did not attend their postpartum visit. This was consistent with the institution’s rate of postpartum loss to follow-up and highlights another potential area for care improvement.

## Conclusions

In this study, patients with adequate prenatal care were not more likely to plan for a very effective or effective method of contraception. Clinicians should include comprehensive contraceptive and family planning in their routine prenatal care and work to ensure women receive their desired method of contraception in the postpartum period to improve health outcomes. A standardized approach to contraceptive counseling that utilizes patient-centered decision-making and patient autonomy is critical. Barriers to obtaining desired contraception are an important area for future research.

Many women chose very effective and effective methods of postpartum contraception; however, there was no statistically significant association between the quality of prenatal care and planned contraception at hospital discharge. However, there are barriers to obtaining all forms of contraception, especially highly effective postpartum contraceptives and this is an important area for future research.

## Data Availability

The datasets used and/ or analyzed during the current study are available from the corresponding author on reasonable request.

## List of abbreviations

WHO: World health organization
ACOG: American College of Obstetricians and Gynecologists
LARC: Long-acting reversible contraceptives
APNCUI: Adequacy of Prenatal Care Utilization Index
IUD: intrauterine device
